# Mastication in Patients with Spinal Muscular Atrophy Types 2 and 3 is Characterized by Abnormal Efficiency, Reduced Endurance, and Fatigue

**DOI:** 10.1007/s00455-021-10351-y

**Published:** 2021-08-15

**Authors:** A. M. B. van der Heul, R. P. A. van Eijk, R. I. Wadman, F. Asselman, I. Cuppen, R. A. J. Nievelstein, E. Gerrits, W. L. van der Pol, L. van den Engel-Hoek

**Affiliations:** 1 Department of Neurology & Neurosurgery, UMC Utrecht Brain Center, University Medical Center Utrecht, Utrecht, The Netherlands; 2 Biostatistics & Research Support, Julius Center for Health Sciences and Primary Care, University Medical Center Utrecht, Utrecht, The Netherlands; 3 Department of Radiology & Nuclear Medicine, Imaging & Oncology Division, University Medical Center Utrecht, Utrecht, The Netherlands; 4 Department of Languages, Literature and Communication, Utrecht Institute of Linguistics OTS, Utrecht University, Utrecht, The Netherlands; 5 Department of Rehabilitation, Donders Center for Neuroscience, Radboud University Medical Center, Nijmegen, The Netherlands

**Keywords:** Spinal muscular atrophy, Mastication, Oral motor function, Deglutition, Deglutition disorders, Survival motor neuron gene

## Abstract

Mastication problems can have a negative impact on the intake of food and quality of life. This cross-sectional study characterizes mastication problems using clinical and instrumental assessments in patients with spinal muscular atrophy (SMA) types 2 and 3 with self-reported bulbar problems. We included 27 patients (aged 13–67 years), 18 with SMA type 2 and 9 patients with SMA type 3 (of whom three were still ambulant) and applied a questionnaire, clinical mastication tests (TOMASS and 6-min mastication test), and muscle ultrasound of the mastication muscles. Non-ambulant patients demonstrated inefficient mastication as reflected by median *z* scores for masticatory cycles (*z* = 1.8), number of swallows (*z* = 4.3) and time needed to finish the cracker (*z* = 3.4), and limited endurance of continuous mastication as demonstrated by the median *z* scores of the 6-min mastication test (*z* = − 1.5). Patients reported increased fatigue directly after the 6-min mastication test as well as 5 min after completing the test (*p* < 0.001; *p* = 0.003). Reduced maximal mouth opening was associated with mastication problems (*p* < 0.001). Muscle ultrasound of the mastication muscles showed an abnormal muscle structure in 90% of both ambulant and non-ambulant patients. This study aims to understand the nature and underlying mechanisms of mastication problems in patients with SMA types 2 and 3 with reported bulbar problems.

**Supplementary Information:**

The online version contains supplementary material available at 10.1007/s00455-021-10351-y.

## Introduction

Hereditary proximal spinal muscular atrophy (SMA) is caused by a loss of function of the survival motor neuron *SMN1* gene and is characterized by loss of alpha-motor neurons in the brainstem and spinal cord. Muscle weakness is most pronounced in axial, respiratory, and proximal muscle groups of the limbs [1]. Severity shows a wide range, from infantile (SMA type 1) and childhood onset (types 2 and 3) to adult onset (type 4). Natural history studies of SMA have shown a progressive decline in motor function and muscle strength in the course of months (type 1) or years (type 2–4) [2, 3].

Effects of brainstem involvement on facial and bulbar functions have been described [4–6]. Fasciculations of the tongue and contractures of the mandibular joint that cause reduced maximal mouth opening are well known clinical characteristics of SMA, but more complex functions such as chewing have not been analyzed in detail despite their importance and relevance for daily life [7–10]. Questionnaire studies have shown that patients frequently report mastication problems, but their prevalence and severity have not been investigated clinically. Chewing problems may increase the risk of inefficient food passage and blockage of the airway and may negatively impact the social function of eating. This may result in avoidance of food and meals and lead to malnutrition [11–13].

In this study, therefore, we aimed to gain further insight into the efficiency and endurance of mastication [14, 15] in patients with SMA types 2 and 3 by means of questionnaires and clinical tests. We used muscle ultrasound to investigate underlying abnormalities of bulbar muscle groups [16].

## Methods

### Participants

We performed a cross-sectional study between August 2018 and August 2019, applying diagnostic criteria of the SMA Consortium for the classification SMA types 2 and 3 [17–19], i.e., onset between the ages of 6 and 18 months and the ability to sit independently at some stage of life for SMA type 2 and onset after the age of 18 months and the ability to walk without support at some stage of life for SMA type 3.

Patients (12 years and older) listed in the Dutch SMA registry who participate in an ongoing population-based study on SMA were approached by letter. Those patients with SMA type 2 or 3 who mentioned bulbar problems (i.e., fatigue or difficulty with chewing; coughing when swallowing liquid or solid foods; food getting stuck in the throat; the need for adaption of food; mealtimes longer than 30 min; tube feeding required) were invited to participate.

None of the patients included in the study were treated with nusinersen, risdiplam, or *AAV*-gene therapy. Six patients used pyridostigmine, but not on the day of the study assessments [20, 21].

The Medical research and Ethics Committee (METC) of the University Medical Center Utrecht approved the study protocol according to the Dutch legislation on clinical studies (METC 17–718). Informed consent was obtained from the patients or from the parents and patients, if they were younger than 16 years.

### Study Design

Mastication was assessed using a combination of a self-report questionnaire, clinical observation, and clinical and instrumental tests (e.g., video fluoroscopic swallowing study and muscle ultrasound). Two experienced speech and language therapists (AMBH and LEH) performed the assessments. One of them carried out the test, while the other video-taped the patient. Outcome scores were based on consensus.

### Questionnaires and Clinical Scales

Bulbar problems were assessed with the Diagnostic List of Dysphagia and Dysarthria in (pediatric) patients with Neuromuscular Diseases (DDD(p)NMD) (see Supplementary file 1). This questionnaire, which has been used previously for patients with SMA [13], consists of 39 yes/no questions and two multiple choice questions about masticatory, swallowing, and jaw issues and consequences for mealtime duration, food adaptations, weight, and the occurrence and frequency of respiratory infections.

We used the functional oral intake scale (FOIS), an ordinal scale that reflects functional oral intake of patients with dysphagia. The scores range from 1 (nothing by mouth) to 7 (total oral diet with no restrictions) [22].

### Clinical Mastication Tests

Efficiency of mastication was assessed using the test of mastication and swallowing solids (TOMASS) [14]. Patients had to eat a single portion of a standardized cracker (4.5 × 4.5 cm) as quickly as possible, but at a safe pace, and to say ‘yes’ when they were finished. We video-taped the patient laterally to determine the number of discrete bites, masticatory cycles (i.e., one cycle is the opening and closing of the jaws), and swallows per cracker, as well as the total time needed to finish the cracker (timed from the moment the cracker passed the bottom lip until the patient indicated that he was finished). Results were compared with normative Dutch data using *z* scores [14, 23].

Endurance of continuous mastication was assessed with the Six-Minute Mastication test (6MMT) [15]. We used this test to capture endurance, which is often reduced in patients with SMA, as shown by a decline in physical performance over a given time of repetitive task performance [24]. For the test, the patient chews continuously on a chewing tube (Theratube©, also known as Thera-tubing©, level 4 for adults), for a period of six minutes. This test was the last of the battery of assessments to avoid any influence on the performance of other tests. We video-taped patients laterally to enable proper visualization of the masticatory movements. We documented the total number of masticatory cycles and the % difference in masticatory cycles between minute 1 and minute 6 as outcome measures for endurance. In addition, we rated rhythm of mastication (rhythmic, variable, not rhythmic) and magnitude of movements (normal, large, or small masticatory movements). To measure the subjective perception of fatigue and pain of masticatory muscles, we used a visual analogue scale (VAS, 0 = no pain, 10 is severe pain; 0 = no fatigue, 10 = severe fatigue) directly after the test and 5 min later. Patients were allowed to see their first score when giving their second score. VAS-scores were compared with the Dutch normative data of the 6MMT [15].

### Active Maximal Mouth Opening and Dental Occlusion

Active (unassisted) maximal mouth opening (aMMO) was assessed with a TheraBite® range of motion scale (Atos Medical AB, Hörby, Sweden). We measured the distance between the mesioincisal angle of the right upper and lower front teeth plus the overbite while the patient opened the mouth as wide as possible. A maximal mouth opening of 40 mm was considered as the lower limit of normal [8, 25]. We documented dental occlusion using a mouth spreader. Occlusal contacts were classified as normal, anterior/posterior open bite or cross bite.

### Muscle Ultrasound

**Fig. 1:**
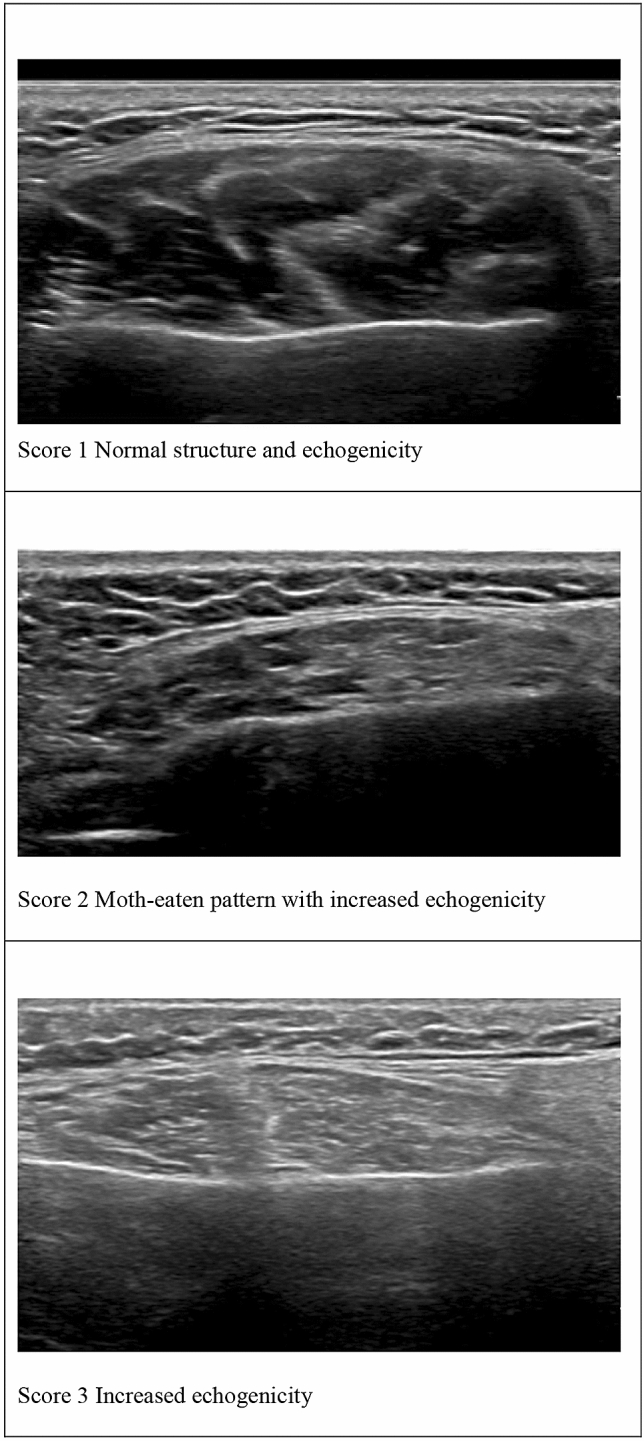
Muscle ultrasound images of the masseter muscle

Muscle ultrasound was performed to visualize the structure of the masticatory muscles (m. masseter and m. temporalis) and tongue muscles using an Affiniti 70 Philips ultrasound system (Philips, the Netherlands) with a 12–5 MHz transducer. For the masseter muscle, the probe was placed on the cheek perpendicular to the jaw line (mandible) with a depth setting of 4 cm. For the temporalis muscle, the probe was placed on the upper border and parallel to the zygomatic arch and moved cranially until the muscle was visible. For the tongue, we used a broadband linear intra operative (so called *hockey stick*) 14–5 MHz transducer [16]. We stored data as DICOM images. The images were scored qualitatively based on consensus (author LEH with another speech and language therapist experienced in muscle ultrasound images) either as ‘normal structure and echogenicity,’ ‘moth-eaten pattern with increased echogenicity’ or ‘increased echogenicity without moth-eaten pattern’ (Fig. 1) [26, 27]. The raters were blind for patient information and did not have access to results of other assessments of the study.

### Statistical Analysis

Categorical variables were specified as number and percentage or median and range. The results of the self-reported mastication problems (questionnaire) and mastication tests were stratified according to the level of motor function: non-ambulant versus ambulant patients.

Results of the TOMASS were presented as *z* scores. The number of bites, mastication cycles, swallows, and total time (in seconds) to eat the cracker were standardized into *z* scores using Dutch normative data, collected from 134 healthy children (4–18 years of age) and 124 healthy adults (20–80 years of age) [28]. *Z* scores of the 6 MMT for total number of mastication cycles, difference in masticatory cycles between minute 1 and minute 6, and VAS-scores for pain and fatigue were obtained based on Dutch normative data, collected from 215 healthy subjects (9–80 years of age) [15]. Median VAS-scores for pain and fatigue of the SMA patients and healthy subjects were compared using a one-sample Wilcoxon signed-rank test.

Associations between the questionnaire (‘do you adapt food’; ‘difficulty with chewing’) and efficiency of mastication (dichotomized *z* scores (< / ≥ 1.5) of the TOMASS’ ‘total time needed to finish the cracker’) were tested using Fisher's exact test for categorical data.

The relation between aMMO and masticatory skills was explored using univariate linear regression. Due the right-skew in TOMASS time, we regressed the aMMO with the natural logarithm of time. Results were translated to the original scale to improve interpretation. A *p*-value of < 0.05 was considered as significant. Data were analyzed using SPSS 25 (IBM Corp. Released 2017. IBM SPSS Statistics for Windows, Version 25.0. Armonk, NY: IBM Corp).

## Results

Patient characteristics are described in Table 1. We included 18 patients with SMA type 2 and 9 patients with SMA type 3 (3 ambulant, 6 non-ambulant).

**Table 1:** Patient characteristics

	SMA type 2 (*n* = 18)	SMA type 3 (*n* = 9)
Sex (F: M)	13:5	6:3
Age in years, median (range)	33 (13–61)	54 (30–67)
Non-ambulant:ambulant	18:0	6:3
Current respiratory status		
No respiratory management, *n* (%)	14 (78)	6 (67)
Non-invasive ventilation, *n* (%)	4 (22)	2 (22)
Invasive ventilation, *n* (%)		1 (11)
aMMO (in mm) non-ambulant patients, median (range)	24 (16–53)	37 (21–43)
aMMO (in mm) ambulant patients, median (range)	n/a	51 (50–55)
Anterior open bite	4 (22)	2 (22)^a^
Posterior open bite	5 (28)	1 (11)
Cross bite	13 (72)	4 (44)
FOIS, median (range)	5 (27)	5 (57)

*F* female, *M* male; *aMMO* active maximal mouth opening, *mm* millimeter, *n/a* not applicable, *FOIS* functional oral intake scale

^a^Dental occlusion of 1 patient missing

### Questionnaire

Twenty-four non-ambulant patients (18 patients with SMA type 2 and 6 patients with SMA type 3) reported difficulty with one or more of the items ‘biting off hard food’ (71%), ‘difficulty with chewing’ (67%), and ‘fatigue while chewing’ (71%). A majority adapted food by pureeing or cutting solid food into small pieces (88%) or reported mealtimes lasting longer than 30 min (54%). Eight patients (33%) reported myalgia, cramp, or tiredness in the jaws when eating or when tired.

The three ambulant patients did not mention difficulty with mastication, difficulty biting off hard food, adapting food, or longer mealtimes. One ambulant patient reported fatigue when chewing.

#### TOMASS

The results of the TOMASS are summarized in Table 2. *Z* scores of < 1.5 (TOMASS) were considered normal. The number of discrete bites of the non-ambulant patients was similar to that of healthy subjects (median *z* score 0.4), but on average, the non-ambulant patients needed more masticatory cycles (median *z* score 1.8), swallows (median *z* score 4.3), and time to finish the cracker (median *z* score 3.4). Efficiency of mastication in non-ambulant patients with SMA type 3 was comparable to patients with SMA type 2 (Supplementary file 2).

**Table 2:** *Z* scores of the TOMASS and 6 MMT for non-ambulant/ambulant patients, and non-ambulant patients with SMA type 2/3

	TOMASS			6MMT		
	Discrete bites	Masticatory cycles	Swallows	Time	Masticatory cycles	Difference M1–M6
SMA types 2 and 3/non-ambulant patients, median (range)	0.4 ( − 1.3 to 1.3)	1.8 (− 1.0 to 8.9)	4.3 ( 0.3 to 13.7)	3.4 (− 0.6 to 12.0)	− 1.5 ( − 3.0—0.3)	− 0.4 ( − 1.7 to 1.0)
SMA type 3/ambulant patients, median (range)	− 0.4 ( − 1.3 to 0.4)	− 0.2 ( − 0.6 to 0.1)	0.3 ( 0)	0.1 ( − 0.1 to 0.5)	− 1.1 ( − 1.7 to − 1.1)	− 0.1 ( − 1.1 to 0.3)
SMA type 2 patients, median (range)	0.4 (− 1.3 to 1.3)	1.8 (− 1.0 to 7.0)	4.8 (0.3 to 13.7)	3.4 (− 0.6 to 12.0)	− 1.5 (− 3.0 to 0)	− 0.4 (− 1.6 to 1.0)
SMA type 3 patients/non-ambulant, median (range)	0.4 (− 0.7 to 1.3)	1.7 (− 0.4 to 8.9)	2.6 (0.3 to 7.0)	2.8 (− 0.1 to 7.9)	− 2.0 (− 3.0 to 0.3)	0.2 (− 1.7 to 0.4)

Outcome measures of the TOMASS: discrete bites: number of bites needed to finish the standardized cracker; masticatory cycles: number of masticatory cycles (i.e., one cycle is the opening and closing of the jaws); swallows: number of observed movements of the thyroid cartilage; time: duration of the time needed to finish the cracker. Outcome measures 6MMT: masticatory cycles: total number of masticatory cycles; difference in masticatory cycles between minute 1 and minute 6

*TOMASS* Test of mastication and swallowing solids, *6MMT* 6-min mastication test

The three ambulant patients performed the test similarly to healthy subjects for discrete bites (median *z* score − 0.4), masticatory cycles (median *z* score − 0.2), swallows (median *z* score 0.3), and time needed to finish the cracker (median *z* score 0.1).

### 6-Min Mastication Test (6MMT)

The results of the 6MMT are summarized in Table 2. *Z* scores of < -1.5 were considered normal. Five non-ambulant patients (21%) could not finish the 6MMT due to excessive fatigue of the masticatory muscles (see Supplementary file 3 for overview). One patient did not want to participate in the test because of jaw symptoms. The rate of chewing tended to be slow (median *z* score − 1.5, range − 3.8 to 0.3) and in four of 18 patients (22%), the difference in masticatory cycles between minute 1 and minute 6 was greater compared to healthy subjects (i.e., *z* scores ≥ − 1.5). One of the three ambulant patients chewed at a slow pace (*z* score -1.7). In all three patients, difference in masticatory cycles between minute 1 and minute 6 were similar to those in healthy subjects.

**Fig. 2:**
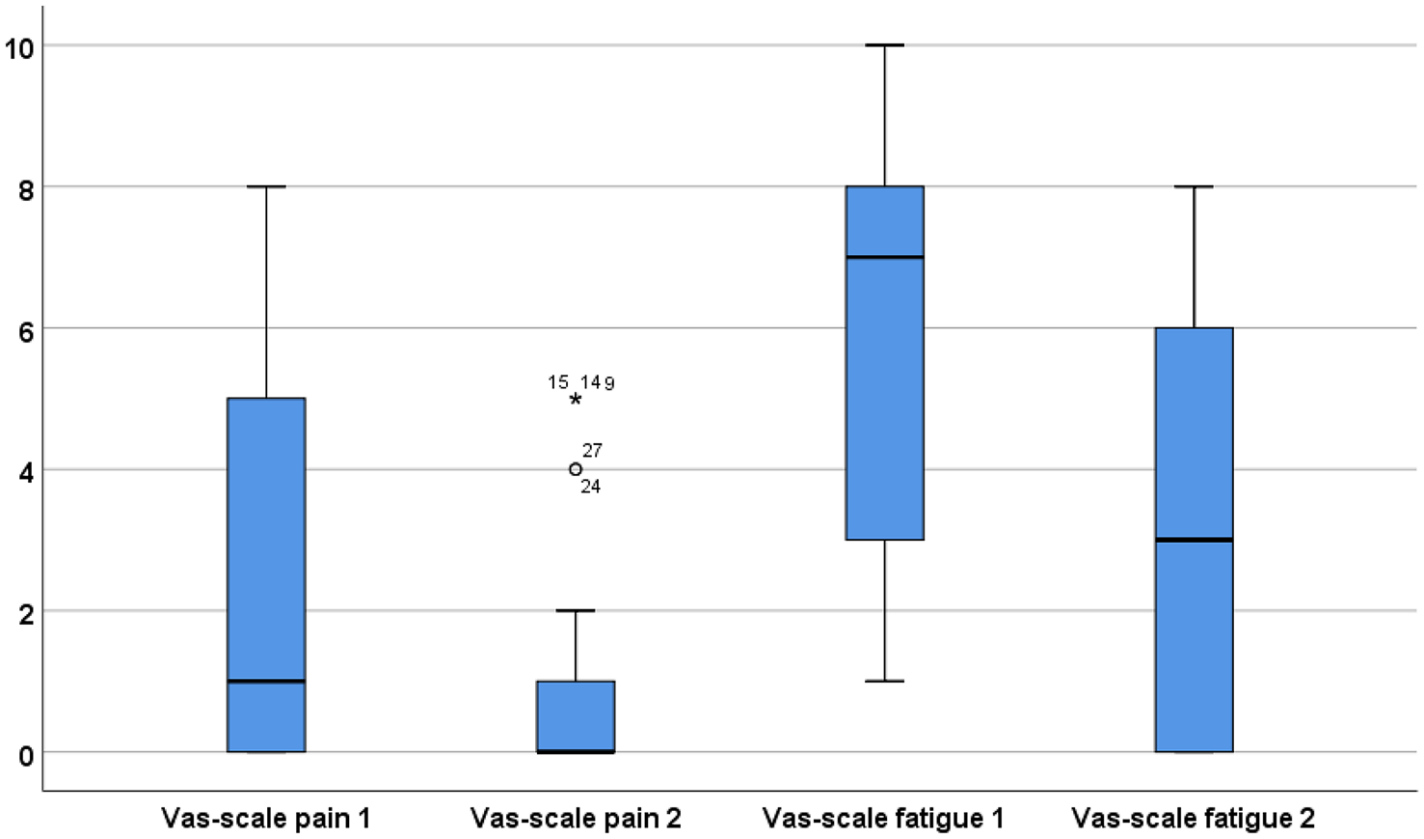
VAS-scales for pain and fatigue after the 6-min mastication test. Distribution of the data of the VAS-scales (0 = no complaints, 10 = severe complaints) for pain directly after the test (pain 1), pain 5 min after the test (pain 2), fatigue directly after the test (fatigue 1), and fatigue 5 min after the test (fatigue 2)

Table 3 summarizes median VAS-scores for pain and fatigue directly after the 6MMT and 5 min later. SMA patients experienced increased pain directly after the test (*p* = 0.037), and 5 min after the test (*p* = 0.011). Patients experienced significantly more fatigue compared to healthy subjects, both directly after the test (*p* < 0.001) and 5 min after the test (*p* < 0.003) (Fig. 2). The VAS-scores for fatigue of the three ambulant patients were 2, 6, and 8 directly after the test; 5 min after the test, they were 0, 6, and 7.

**Table 3:** Median VAS-scores of the 6MMT, directly after the test (pain I, fatigue I) and 5 min after the test (pain II, fatigue II)

	Non-ambulant patients (*n* = 24)	Ambulant patients (*n* = 3)
VAS-score pain I	1 (range 0–8)	1 (range 0–5)
VAS-score pain II	0 (range 0–5)	0 (range 0–4)
VAS-score fatigue I	7 (range 1–10)	6 (range 2–8)
VAS-score fatigue II	2.5 (range 0–8)	6 (range 0–7)

The median VAS-score of healthy subjects (*n* = 153) was for pain 1 (range 0–7) directly after the test (pain I) and 0 (range 0–6) 5 min after the test (pain II). For fatigue, the median VAS-score was 2 (range 0–9) directly after the test (fatigue I) and 1 (range 0–6) 5 min after the test (fatigue II)

Fifteen of 23 non-ambulant patients (65%) had rhythmical jaw movements during mastication, 4 patients (17%) showed variable movements, and 4 patients (17%) had arrhythmical jaw movements. Magnitude of jaw movements was qualified as normal in 4 patients (17%) and small in 19 patients (83%). There were no patients with large jaw movements. The three ambulant patients had rhythmical jaw movements of normal magnitude.

### Relation Between Active Maximal Mouth Opening and Results of the TOMASS

**Fig. 3:**
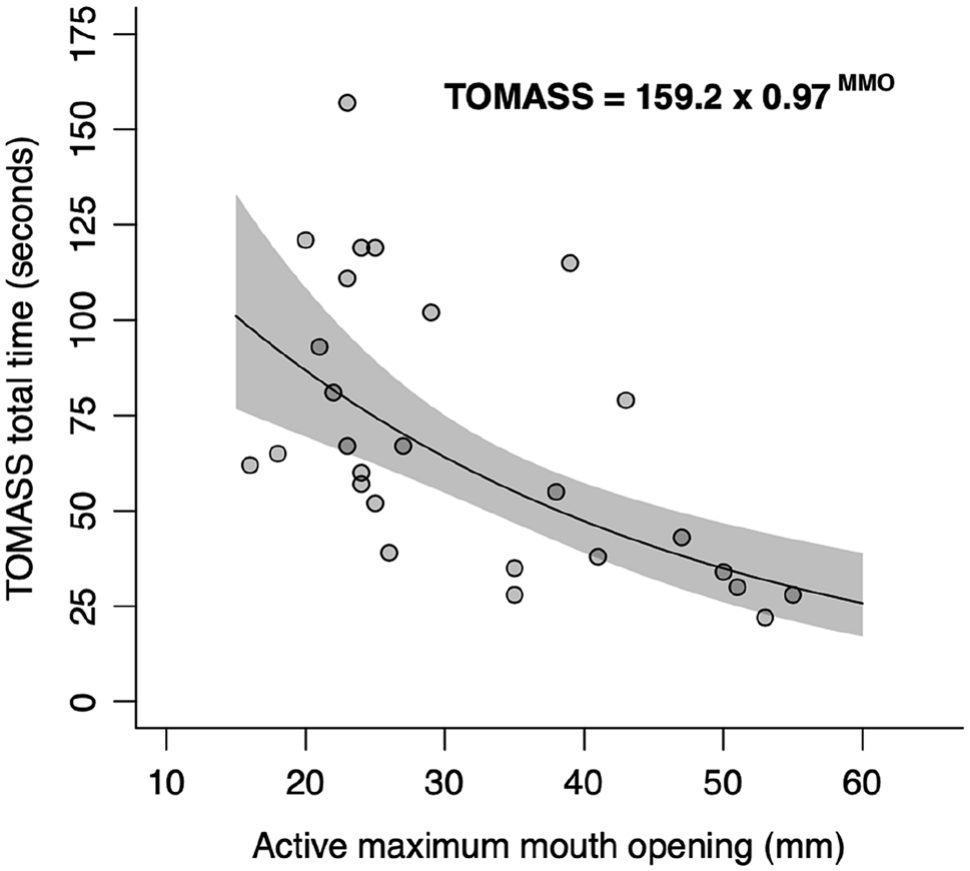
Linear regression between the log (TOMASS total time) and active maximal mouth opening. Per mm increase in active maximal mouth opening, the TOMASS total time decreases by 3% (95% CI 1.60 to 4.36%, *p* < 0.001)

Univariate regression analysis showed that aMMO significantly contributed to the time needed to finish a standardized cracker (*F* (1,25) = 19.28, *p* < 0.001) (Fig. 3). The mean effect of an increase of 1 mm in aMMO was a 3% decrease in time needed to finish the cracker (95% CI 1.60 to 4.36). Overall, aMMO explained over 40% of the variation in the log TOMASS total time (adjusted *R*^2^ = 0.413).

### Relation Between Questionnaire and Mastication Test Results

The association between ‘do you adapt food?’ and efficiency of mastication was significant (*p* < 0.001). All six patients who did not adapt food (for instance by cutting into pieces, pureeing it or avoiding hard foods) had normal efficiency of mastication (*z* scores < 1.5). There was also a significant association between ‘do you experience difficulty with chewing?’ and efficiency of mastication (*p* = 0.001). Not all patients were aware of mastication problems because three of 11 patients (27%) reported no difficulty chewing, but their efficiency of mastication was abnormal.

### Muscle Ultrasound of Bulbar Muscles

Masticatory muscles frequently showed increased echogenicity with a moth-eaten pattern in non-ambulant and ambulant patients (Fig. 1). The masseter muscle was most often affected (85% of the patients), followed by the temporalis muscle (63% of the patients). Assessments of the tongue muscles were possible in 20 of 27 patients. Ten patients had increased echogenicity with a moth-eaten pattern and 9 patients increased echogenicity of the tongue muscles. In seven of 27 patients (26%), the transducer could not be placed on the tongue as a result of reduced mouth opening. The echogenicity of the tongue muscles could not be determined in these patients.

## Discussion

This study shows that mastication in non-ambulant patients with SMA types 2 and 3 is characterized by a combination of inefficient mastication, reduced endurance, fatigue during and after mastication, and altered oral anatomy (i.e., reduced maximal mouth opening and dental malocclusion). The three ambulant patients not only showed normal masticatory efficiency but also demonstrated reduced endurance or fatigue.

Previous studies have shown brainstem alpha-motor neuron involvement with a caudal-to-rostral gradient in SMA [4–6]. Granger et al. were the first to report reduced bite force, jaw movements, and endurance in 15 patients with SMA [7]. These early observations were corroborated by a small number of studies assessing maximal mouth opening, jaw mobility, and bite force [8–10]. Reduced maximal mouth opening is very common among patients with SMA type 2 [8–10, 29] and probably progresses over time from an early age. MR images show that this complication is caused by the preferential fatty degeneration and atrophy of the lateral pterygoid muscle, which not only mediates mouth opening but also allows the horizontal masticatory movements that increase masticatory efficiency [30].

The first important observation of our study is that non-ambulant patients require more masticatory cycles and a prolonged time before they are ready to swallow. Although this may be attributable to weakness of the masticatory muscles, reduced mobility of the jaws (i.e., horizontal mastication movements) probably has a greater impact. Dental malocclusion which is related to weak masticatory and facial muscles may further aggravate masticatory inefficiency [31].

A second important observation was reduced endurance of continuous mastication. Limited endurance (i.e. ‘fatigability’) is a specific feature of SMA during repetitive muscle contractions and may be at least partially caused by abnormal neuromuscular junction function [2, 21, 24, 32, 33].

Ultrasound showed increased echogenicity of masseter and temporalis muscles both in non-ambulant and ambulant patients, suggesting widespread abnormalities in bulbar muscles and complementing a previous MRI study [10]. It is the first time that muscle ultrasound of bulbar muscles is performed in SMA patients with documented mastication and/or swallowing problems. The ultrasound images were assessed qualitatively. The specific structure of the muscles previously described for skeletal muscles of patients with SMA was also present in the bulbar muscles of the patients in our study [26, 34]. Despite abnormalities in bulbar muscle structure, ambulant patients still managed to chew efficiently, suggesting functional reserve capacity of muscle force. This is illustrated by the fact that masticatory performance did not differ between patients with SMA and healthy subjects, although a significantly lower maximum voluntary bite force has been reported in this patient group [9]. This phenomenon of a changing muscle structure in combination with maintenance of relatively efficient mastication has also been observed in patients with Duchenne muscular dystrophy [35].

Problems with mastication have a profound impact on the quality of life of patients with SMA. Despite the frequent food adaptations as reflected in the functional oral intake scale (Table 1), patients needed more time to process food compared to healthy subjects. This probably also affects the social function of eating because patients need to stay focused on the task of chewing, while avoiding conversations during mealtime in order to thoroughly prepare the food for safe swallowing.

When comparing questionnaire and clinical test results, we found underreporting of masticatory problems. A considerable number of patients had inefficient mastication but did not report symptoms. This probably reflects the continuous adaptations to the consequences of progressive bulbar problems. It indicates that it is not sufficient to use only a questionnaire to detect mastication problems. Questionnaires combined with aMMO, TOMASS and 6MMT provide quantitative and qualitative information about masticatory performance and can be used in clinical practice. Oral muscle ultrasound is supplemental to the clinical assessment of mastication and may support the explanation of the change in mastication function.

In order to prevent loss of function of masticatory muscles due to disuse, patients are probably best advised to continue to use food they can still chew and swallow safely. Obviously, patients with severe mastication problems will need to adapt the ratio between chewable food and soft food. Although there is little evidence that stretching exercises reverse or delay limitations of aMMO, some patients claimed that stretching stabilized aMMO [36]. Given the major effect of aMMO on chewing efficiency, the effects of stretching need to be studied in more detail.

This study has certain limitations. First of all, the sample size in particular of ambulant patients was small. This can be attributed to the inclusion criterion of having bulbar problems. Although the participation of more ambulant patients would have allowed more definite conclusions, a previous study showed that aMMO in ambulant patients remains within the normal range [9]. This suggests that mastication problems (masticatory inefficiency) are less likely to occur in ambulant patients. Another limitation is that ‘number of swallows’ (TOMASS) were determined by visual observations and not by objective methods such as sEMG.

## Conclusion

Mastication problems in patients with SMA types 2 and 3 are characterized by inefficiency, reduced endurance, and fatigue, probably caused by masticatory muscle changes that can be detected by ultrasound. Not all patients were aware of their mastication problems. In the future, it would probably be advisable to apply a combination of quantitative and qualitative mastication tests in addition to a questionnaire to test masticatory function in non-ambulant patients with SMA. Interventions should aim to maintain jaw mobility, dental occlusion, and endurance of mastication.

## Supplementary Information

Below is the link to the electronic supplementary material.

Supplementary file1 (PDF 98 kb)

Supplementary file2 (PDF 274 kb)

Supplementary file3 (PDF 147 kb)
