# Supplementary material for: Mastication in Patients with Spinal Muscular Atrophy Types 2 and 3 is Characterized by Abnormal Efficiency, Reduced Endurance, and Fatigue
Source: Dysphagia. 2021 Aug 15;37(4):715–23. doi: 10.1007/s00455-021-10351-y (PMC9345836; doi:10.1007/s00455-021-10351-y)
Supplement: Supplementary file 1 — Supplementary file1 (PDF 98 kb) [file 455_2021_10351_MOESM1_ESM.pdf]

Diagnostic list for Dysphagia and Dysarthria in (pediatric) Patients with Neuromuscular Diseases (DDD(p)NMD)

Are you often ill as a result of swallowing problems ? Yes - No

Do you suffer from respiratory infections? Yes - No

Do you use antibiotics on a long-term basis? Yes - No

Do specific foods cause problems with swallowing? Yes - No

Swallowing problems:

- ☐ never occur;
- ☐ occur with solid foods;
- ☐ occur with pureed food and thick liquids;
- ☐ occur both with liquids and solid foods.

Do you dread mealtimes? Yes - No

Do you sometimes refuse food? Yes - No

Do you adapt food, for instance by cutting it into small pieces, pureeing it or avoiding hard foods? Yes - No

Do you experience difficulty with chewing? Yes - No

Do your jaws get tired when chewing? Yes - No

Do mealtimes take a long time (longer than 30 minutes)? Yes - No

Do you tend to choke (‘yes’ if you choke more than once a day) Yes – No

Do you tend to gag or vomit? Yes – No

Do you cough when swallowing solid foods? Yes – No

Do you cough when swallowing liquids? Yes - No

Do you feel as if food is sticking in your throat? Yes - No

The feeling of food sticking in the throat:

- □ Does not occur;
- □ Occurs with solid foods;
- □ Occurs with pureed and thick liquids;
- □ Occurs with both liquids and solid foods.

Do you experience excessive burping? Yes - No

Do you get tired when eating? Yes - No

When you eat do you tend to drool? Yes - No

Is your mouth often open? Yes - No

Is your mouth opening limited for brushing teeth? Yes - No

Do you experience jaw problems with biting off a large piece of food? Yes – No

Do you experience jaw problems with biting off hard foods ? Yes – No

Do you experience jaw problems when yawning? Yes – No

Do you experience jaw problems when laughing? Yes – No

Do you experience jaw problems when you are tired? Yes – No

Do you experience jaw problems when you are waking up? Yes – No

Is your daily intake for solid foods sufficient? Yes - No

Is your daily intake of liquids sufficient? Yes - No

Do you make (partial) use of tube feeding? Yes - No

Are you gaining too much weight? Yes - No

Are you losing weight? Yes - No

Is your weight too low Yes - No

Is (or was) a dietician involved?	Yes - No
Do you suffer from shortness of breath when talking?	Yes – No
Do you get tired when talking?	Yes – No
When you talk to people, do they ask you to repeat what you said?	Yes – No
Do people have problems understanding you when you are talking in a noisy environment?	Yes – No
Do you suffer from a sore throat due to talking?	Yes – No
Is your voice loud enough?	Yes - No
