# Supplementary material for: Mastication in Patients with Spinal Muscular Atrophy Types 2 and 3 is Characterized by Abnormal Efficiency, Reduced Endurance, and Fatigue
Source: Dysphagia. 2021 Aug 15;37(4):715–23. doi: 10.1007/s00455-021-10351-y (PMC9345836; doi:10.1007/s00455-021-10351-y)

Supplementary file 2

Paper: **Mastication in patients with spinal muscular atrophy types 2 and 3 is characterized by abnormal efficiency, reduced endurance and fatigue**

Journal: **Dysphagia**

Authors: **A.M.B. van der Heul, MSc[1], R.P.A. van Eijk[1,2], MD, PhD, R.I. Wadman, MD, PhD[1], F. Asselman, MSc[1], I. Cuppen, MD, PhD[1], R.A.J. Nievelstein MD, PhD[3], E. Gerrits, PhD[4], W.L. van der Pol, MD, PhD[1]*, L. van den Engel-Hoek, PhD[5]***

Z-scores of the TOMASS for patients with SMA type 2 and 3. A: discrete bites; B: masticatory cycles; C: swallows; D: total time needed to finish the cracker. The circles refer to ambulant patients

A. Discrete bites

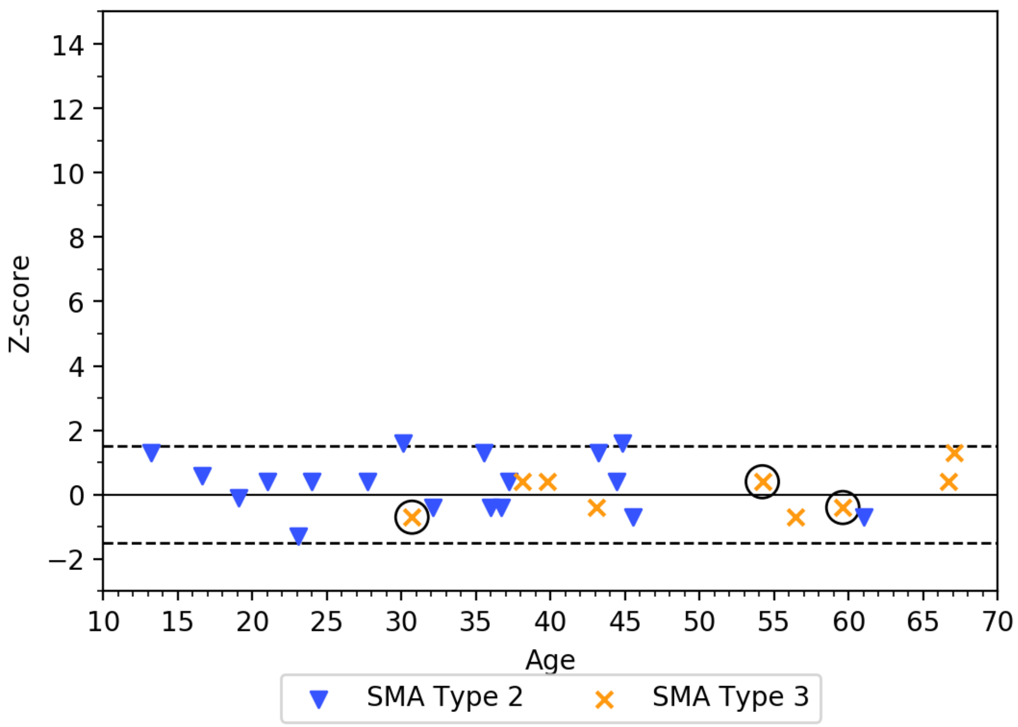

B. Masticatory cycles

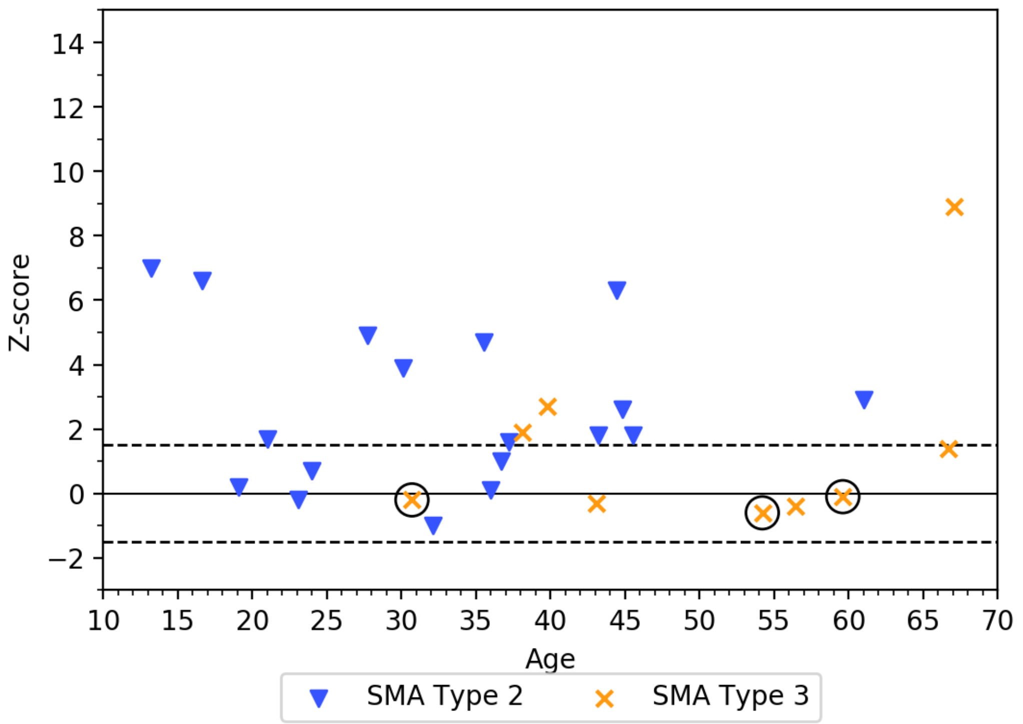

C. Swallows

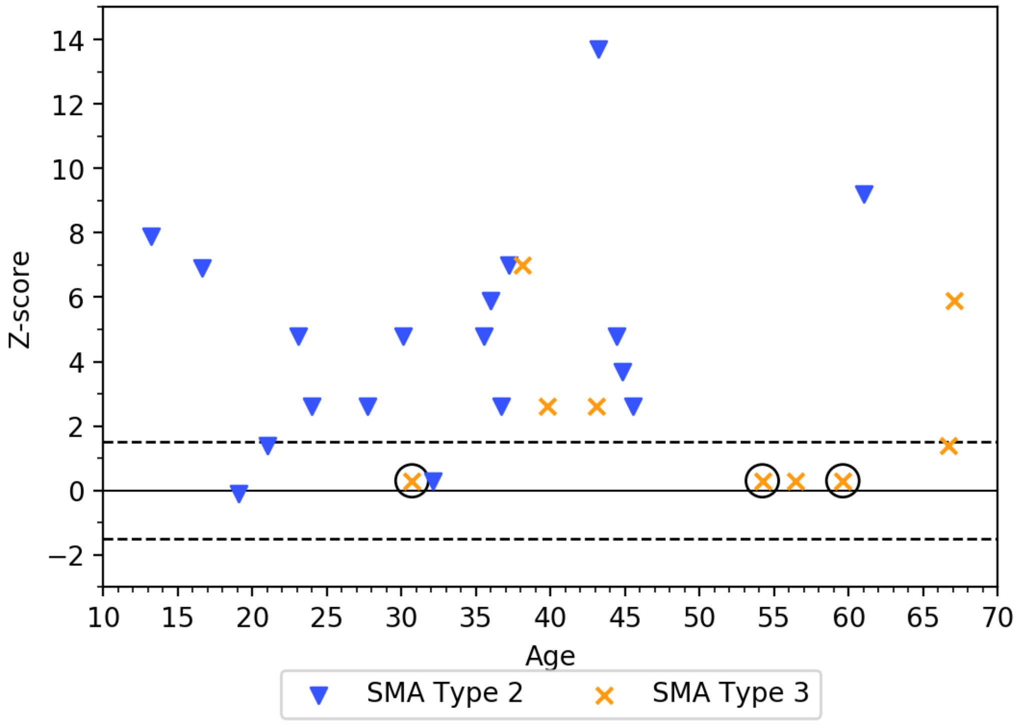

D. Time

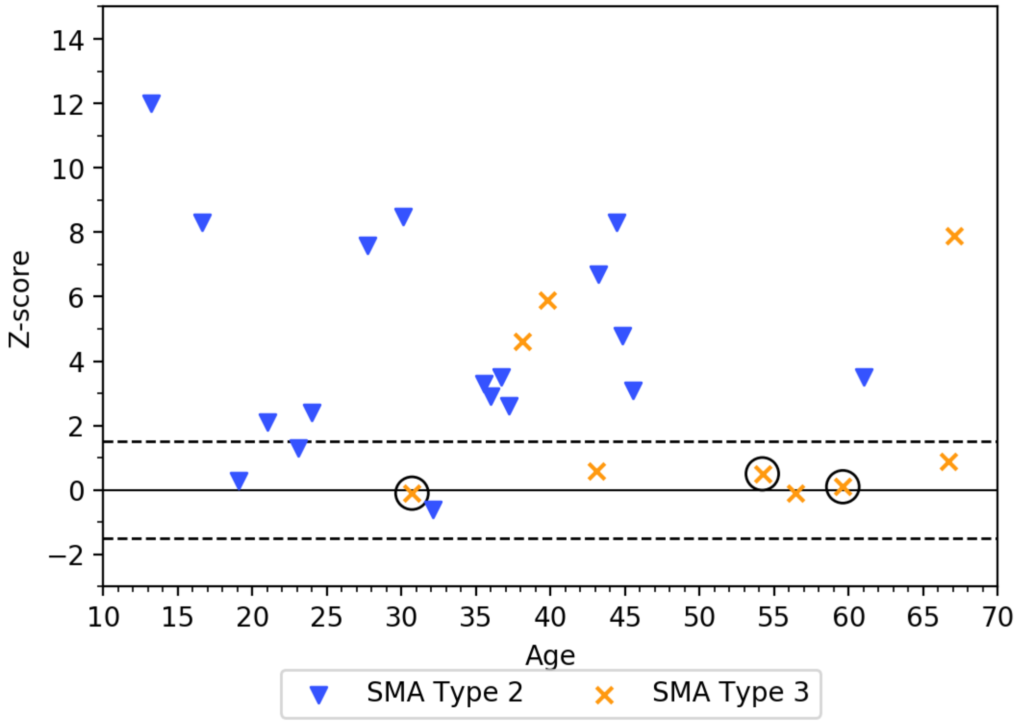

Supplement: Supplementary file 2 — Supplementary file2 (PDF 274 kb) [file 455_2021_10351_MOESM2_ESM.pdf]
