# Supplementary material for: Mastication in Patients with Spinal Muscular Atrophy Types 2 and 3 is Characterized by Abnormal Efficiency, Reduced Endurance, and Fatigue
Source: Dysphagia. 2021 Aug 15;37(4):715–23. doi: 10.1007/s00455-021-10351-y (PMC9345836; doi:10.1007/s00455-021-10351-y)
Supplement: Supplementary file 3 — Supplementary file3 (PDF 147 kb) [file 455_2021_10351_MOESM3_ESM.pdf]

Supplementary file 3

Paper: **Mastication in patients with spinal muscular atrophy types 2 and 3 is characterized by abnormal efficiency, reduced endurance and fatigue**

Journal: **Dysphagia**

Authors: **A.M.B. van der Heul, MSc[1], R.P.A. van Eijk[1,2], MD, PhD, R.I. Wadman, MD, PhD[1], F. Asselman, MSc[1], I. Cuppen, MD, PhD[1], R.A.J. Nievelstein MD, PhD[3], E. Gerrits, PhD[4], W.L. van der Pol, MD, PhD[1]*, L. van den Engel-Hoek, PhD[5]***

Z-scores of the TOMASS and 6 MMT for each patient (n=27)

			TOMASS				6MMT	
	Age	SMA type	Discrete bites	Masticatory cycles	Swallows	Time	Masticatory cycles	Difference M1 – M6
1	13	2	1.3	7.0	7.9	12.0	-2.2	-0.3
2	16	2	0.1	6.6	6.9	8.3	-1.8	-0.4
3	19	2	-0.4	0.5	0.3	0.9	-0.4	-0.1
4	21	2	0.4	1.7	1.4	2.1	-1.9	-1.3
5	23	2	-1.3	-0.2	4.8	1.3	-0.5	0.9
6	24	2	0.4	0.7	2.6	2.4	-3.0	[1]
7	27	2	0.4	4.9	2.6	7.6	-1.5	-1.5
8	30	2	0,4	3,9	4,8	8,5	-1,5	-1,0
9	32	2	-0.3	-1.0	0.3	-0.6	-1.3	-0.2
10	35	2	1.3	4.7	4.8	3.3	-0.8	-1.5
11	36	2	-0.4	0.1	5.9	2.9	-0.8	1.0
12	36	2	-0.4	1.0	2.6	3.5	-1.8	-1.6

13	37	2	0.4	1.6	7.0	2.6	0	-1.3
14	43	2	1.3	1.8	13.7	6.7	-1.0	-0.2
15	44	2	0.4	6.3	4.8	8.3	-3.0	[1]
16	44	2	0,4	2.6	3.7	4.8	-1.6	-0.1
17	45	2	-0.7	1.8	2.6	3.1	-3.0	[1]
18	61	2	-0.7	2.9	9.2	3.5	-0.9	-0.6
**19**	**30**	**3**	**-1,3**	**-0.2**	**0.3**	**-0.1**	**-1.7**	**-0.1**
20	38	3	0.4	1.9	7.0	4.6	[2]	[2]
21	39	3	0.4	2.7	2.6	5.9	-3.0	[1]
22	43	3	-0.4	-0.3	2.6	0.6	-2.0	-1.7
**23**	**54**	**3**	**0.4**	**-0.6**	**0.3**	**0.5**	**-1.1**	**0.3**
24	56	3	-0.7	-0.4	0.3	-0.1	0.3	0.2
**25**	**59**	**3**	**-0.4**	**-0.1**	**0.3**	**0.1**	**-1.1**	**-1.1**
26	66	3	0.4	1.4	1.4	0.9	0.1	0.4
27	67	3	1.3	8.9	5.9	7.9	-3.0	[1]

TOMASS = test of mastication and swallowing solids; 6MMT = 6 minutes mastication test; Difference M1 – M6 = difference between minute 1 and minute 6

[1] patient could not finish the test, [2] test was not performed because of jaw-complaints

The bold data refer to ambulant patients

The shaded areas are patients with posterior open bites
